# Age-related changes in tissue macrophages precede cardiac functional impairment

**DOI:** 10.18632/aging.100669

**Published:** 2014-05-23

**Authors:** Alexander R. Pinto, James W. Godwin, Anjana Chandran, Lucy Hersey, Alexei Ilinykh, Ryan Debuque, Lina Wang, Nadia A. Rosenthal

**Affiliations:** ^1^Australian Regenerative Medicine Institute (ARMI), Monash University, Clayton, Victoria 3800, Australia; ^2^National Heart and Lung Institute, Imperial College London, UK

**Keywords:** Cardiac macrophages, inflammation, cardiac senescence, Cx3cr1, tissue macrophages, ageing

## Abstract

Cardiac tissue macrophages (cTMs) are abundant in the murine heart but the extent to which the cTM phenotype changes with age is unknown. This study characterizes aging-dependent phenotypic changes in cTM subsets. Using the *Cx_3_cr1^GFP/+^* mouse reporter line where GFP marks cTMs, and the tissue macrophage marker Mrc1, we show that two major cardiac tissue macrophage subsets, Mrc1^−^GFP^hi^ and Mrc1^+^GFP^hi^ cTMs, are present in the young (<10 week old) mouse heart, and a third subset, Mrc1^+^GFP^lo^, comprises ~50% of total Mrc1^+^ cTMs from 30 weeks of age. Immunostaining and functional assays show that Mrc1^+^ cTMs are the principal myeloid sentinels in the mouse heart and that they retain proliferative capacity throughout life. Gene expression profiles of the two Mrc1^+^ subsets also reveal that Mrc1^+^GFP^lo^ cTMs have a decreased number of immune response genes (Cx_3_cr1, Lpar6, CD9, Cxcr4, Itga6 and Tgfβr1), and an increased number of fibrogenic genes (Ltc4s, Retnla, Fgfr1, Mmp9 and Ccl24), consistent with a potential role for cTMs in cardiac fibrosis. These findings identify early age-dependent gene expression changes in cTMs, with significant implications for cardiac tissue injury responses and aging-associated cardiac fibrosis.

## INTRODUCTION

Mononuclear phagocytes (MPs), including dendritic cells (DCs) and macrophages play an important role in tissue homeostasis by serving as sentinels for damage and foreign antigens, but are highly heterogeneous depending on tissue location and environment. Using the *Cx_3_cr1^GFP/+^* transgenic mouse line, in which GFP specifically labels monocytes, macrophages and dendritic cells, we recently reported that cardiac tissue macrophages (cTMs) comprise a distinct set of tissue macrophages [1], closely resembling alternatively-activated ‘M2’ macrophages detected at the late phases of tissue injury [2]. Consistent with their M2-like signature, cTMs express many genes involved in the dampening of local tissue inflammation such as interleukin 10 (IL-10), insulin-like growth factor 1 (IGF-1) and complement component 1q (C1q) [1].

While cTMs have been characterized in the context of the resting heart, the extent to which age-dependent changes occur in cTMs and the potential implications of these changes for cTM injury responses has not been previously examined. A number of studies have characterized cells involved in the post-injury cardiac inflammatory response. The role of MPs in the injured heart has principally been examined from the perspective of infiltrating mononuclear cells that transiently differentiate into macrophages at the tissue injury lesion [3]. Yet, as in many other tissues [4], the role of endogenous modulators of cardiac inflammation, such as cTMs, is not fully understood.

The self-renewing capacity of cTMs with aging is an additional facet of their biology that requires further study. Recent reports have indicated that tissue macrophages have the capacity to renew by local proliferation [5]. Indeed, macrophages have been reported to proliferate locally in response to inflammatory stimuli [6, 7]. However whether cTM local self-renewal capacity persists with aging and senescence is unknown.

In this study we examined age-related changes in cTM gene expression and the potential impact of these changes on cardiac injury responses. We determined that cTMs proliferate *in situ*, and retain proliferative capacity throughout life. After birth, cTMs lose a number of chemokine and injury response receptors, with the greatest change occurring in epicardial cTMs. The reduced expression of these receptors has significant implications for age-related decrements in cardiac injury responses, whilst up-regulation of profibrotic genes accompanies cardiac senescence. These findings provide new insights into age-dependent changes in macrophage function within the mouse heart, underscoring their potential significance for tissue damage responses, inflammatory modulation, and senescence.

## RESULTS

### Cx_3_cr1 gene expression in Mrc1^+^ cTMs is lost with age

**Figure 1:**
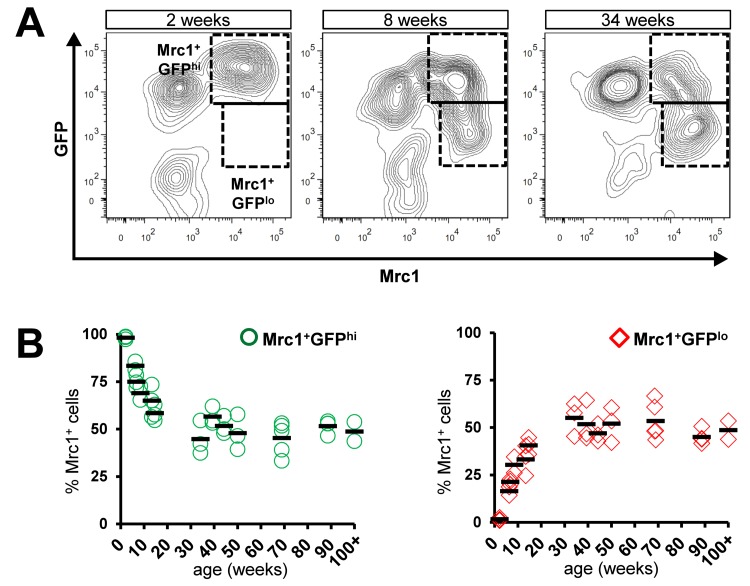
Age-dependent decline in proportion of Mrc1^+^ cTMs expressing high levels of Cx_3_cr1. **(A)** Representative contour scatter plots of GFP^+^ and/or Mrc1^+^ cTMs from 2, 8 or 34 week old *Cx_3_cr1^GFP/+^*mouse hearts (left, middle and right panels respectively). Dotted boxes demarcate Mrc1^+^GFP^hi^ and Mrc1^+^GFP^lo^ cTMs. **(B)** Decrease in proportion of Mrc1^+^GFP^hi^ cTMs (O) and increase in Mrc1^+^GFP^lo^ cTMs (⋄) with age (left and right panels, respectively)

In a previous study, we characterized a dense population of cTMs identified by GFP expression in *Cx_3_cr1^GFP/+^*mouse hearts [1]. A subset of these cells also express the scavenger receptor Mrc1, which is commonly found on alternatively-activated ‘M2’ macrophages, as well as other M2 macrophage-related genes [1]. To detect age-related changes in *Cx_3_cr1* expression in the Mrc1^+^ subsets, we conducted flow cytometric analysis of cardiac myeloid cells (CD45^+^CD11b^+^ cells) prepared from *Cx_3_cr1^GFP/+^* mouse hearts, ranging in age from 2 to 100 weeks (Fig. 1). In addition to Mrc1^−^GFP^hi^ and Mrc1^+^GFP^hi^ cells, a new subset of cTMs emerged with age, marked by low Cx_3_cr1 (GFP) expression within the Mrc1^+^ cTMs (Mrc1^+^GFP^lo^), with GFP levels declining most rapidly between 0 and 12 weeks (Fig. 1B). In contrast, hearts from mice ranging in age from 34 to 100 weeks had a stable proportion of Cx_3_cr1 expressing cells within Mrc1^+^ cTMs (Fig. 1B).

To determine whether GFP or Mrc1 expression predominantly marks myeloid leukocytes in *Cx_3_cr1^GFP/+^* mouse hearts, we gated viable cells (viability dye^−^) and determined GFP or Mrc1 expression relative to CD45 or CD11b expression (Supplemental Fig. 1. The vast majority of both GFP^+^ and Mrc1^+^ cells were CD45^+^ and CD11b^+^, indicating that GFP^+^ and Mrc1^+^ cells within *Cx_3_cr1^GFP/+^* mouse hearts are predominantly myeloid leukocytes.

### Functional heterogeneity of cTMs

**Figure 2:**
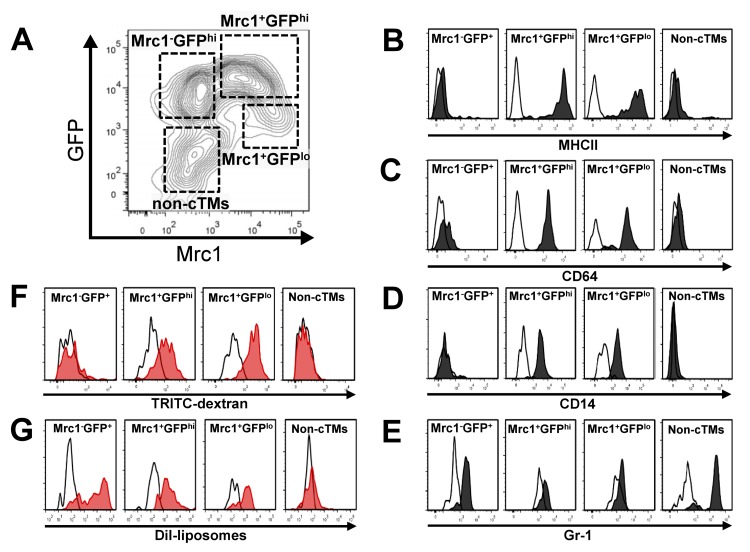
Immunophenotypic and functional characteristics of cTMs. (A) Contour scatter plot of cardiac myeloid cells (CD45^+^CD11b^+^) from *Cx_3_cr1^GFP/+^* mouse hearts with GFP^+^ and/or Mrc1^+^ cTMs in addition to non-cTMs (Mrc1^−^GFP^−^) demarcated. Flow cytometry histogram of cell surface staining of cTM subsets and non-cTMs for MHCII **(B)**, CD64 **(C)**, CD14 **(D)** and Gr-1 **(E)**. Flow cytometry histogram of 2 MDa TRITC-labelled dextran **(F)** or DiI-liposome uptake **(G)** of cTM subset and non-cTMs. White filled histograms represent signals from isotype controls (**B-E**) or uninjected mice (**F** and **G**)

Cell surface antigen profiling confirmed the macrophage identity of cTM subsets and non-cTM myeloid cells in the mouse heart (Fig. 2A). Mrc1^+^ cells expressed high levels of MHCII whereas Mrc1^−^ cells and non-cTMs were mostly negative for this cell surface marker (Fig. 2B). Similarly, cluster differentiation 64 (CD64) and cluster differentiation 14 (CD14; Fcγ receptor 1 and lipopolysaccharide receptors, respectively) signals were detectable in Mrc1^+^ cells, and were absent in Mrc1^−^ and non-cTM cells (Fig. 2C-D). Thus, Mrc1^+^ cells have a profile consistent with tissue macrophages, whereas Mrc1^−^GFP^hi^ cells resemble monocyte-like cells consistent with the absence of MHCII staining [8]. This was supported by the comparison of the side- and forward-scatter profile of cTMs (Supplemental Fig. 2). The monocyte/macrophage identity of Mrc1^+^ and GFP^hi/lo^ cells was supported by negative or low staining for Gr-1 antigen, whereas non-cTM cells stained highly for this antigen (Fig. 2E), suggesting a granulocyte identity. Indeed, non-cTMs did not stain for MHCII, CD64 or CD14 and had a high side- and forward-scatter (Fig. 2 B-E, Supplemental Fig. 2).

The monocyte/macrophage identity of cardiac myeloid cells was further verified by *in vivo* macropinocytic and phagocytic experiments. Injection of Tetramethylrhodamine (TRITC)-labelled high molecular weight (2 MDa dextran) dextran (TRITC-dextran) intravenously into *Cx_3_cr1^GFP/+^* mice was followed by flow cytometric analysis at 20 hours to detect TRITC^+^ cardiac myeloid cells. Both Mrc1^+^ cTM subsets (Mrc1^+^GFP^hi^ and Mrc1^+^GFP^lo^) had extensive macropinocytic activity (Fig. 2F), whereas no dextran uptake was detectable in Mrc1^−^GFP^hi^ cTMs or non-cTMs (Fig. 2F). As a functional marker of MPs, macropinocytosis is a principle mode of antigen detection [9] and a major mechanism whereby apoptotic and necrotic cells can be cleared [10, 11]. Thus, macropinocytic activity implicates Mrc1^+^ cells as the principal cTM subset undertaking extensive environmental surveillance of cardiac tissue for tissue damage and foreign antigen signals.

To determine the phagocytic potential of the three cTM subsets, we injected DiI loaded liposomes [12] intravenously into *Cx_3_cr1^GFP/+^*mice, followed by flow cytometric analysis at 24 hours to detect DiI^+^ cardiac myeloid cells. In contrast to their variable macropinocytic phenotype, all three cTM subsets demonstrated phagocytic activity Fig. 2G), with a higher DiI signal peak in Mrc1^−^GFP^hi^ cTMs compared to Mrc1^+^ cTMs. Low or no DiI signal was detectable in non-cTMs (Fig. 2G). In summary, although all cTM subsets are phagocytic, only Mrc1^+^ cTMs actively sample the local environment by macropinocytosis for damage and foreign antigen signatures. These findings are also consistent with the presence of canonical macrophage markers CD14, CD64 and the professional antigen presenting cell marker MHCII exclusively on Mrc1^+^ cTMs.

### Age-related changes in cTM proliferative capacity and distribution

**Figure 3:**
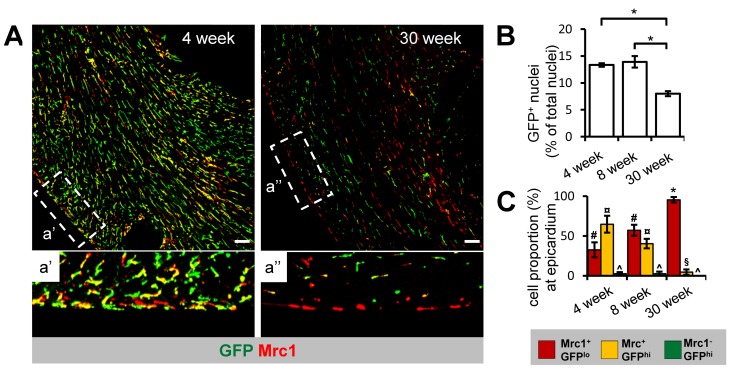
Age-dependent decline in Cx_3_cr1^+^ cTM cell density and proportion at the epicardium **(A)** 45 μm confocal micrograph maximum intensity view of GFP and Mrc1 staining in mouse hearts sections from 4 and 30 week old *Cx_3_cr1^GFP/+^* mouse heart sections (scale bar indicates 100 μm). a' and a'' are magnified views of areas demarked by dotted lines. **(B)** Histogram of proportion of GFP^+^ nuclei in relation to total nuclei in hearts of 4, 8 and 30 week old *Cx_3_cr1^GFP/+^*mouse heart sections. *p≤0.05. **(C)** Histogram of proportion of Mrc1^+^GFP^lo^, Mrc1^+^GFP^hi^ or Mrc1^−^GFP^hi^ cTMs in relation to all cTMs at the epicardium. #*p*>0.05 Mrc1^+^GFP^hi^ vs Mrc1^+^GFP^lo^; ¤*p*≤0.05 Mrc1^−^GFP^hi^ vs Mrc1^+^GFP^hi^; ^*p*≤0.05 Mrc1^−^GFP^hi^ vs Mrc1^+^GFP^lo^; *p≤0.05 Mrc1^+^GFP^hi^ vs Mrc1^+^GFP^lo^*; p*>0.05 Mrc1^−^GFP^hi^ vs Mrc1^+^GFP^hi^. All histograms show means ± SEM. Cell numbers were quantified from at least 4 fields of view from 3 mouse hearts/age group

**Figure 4:**
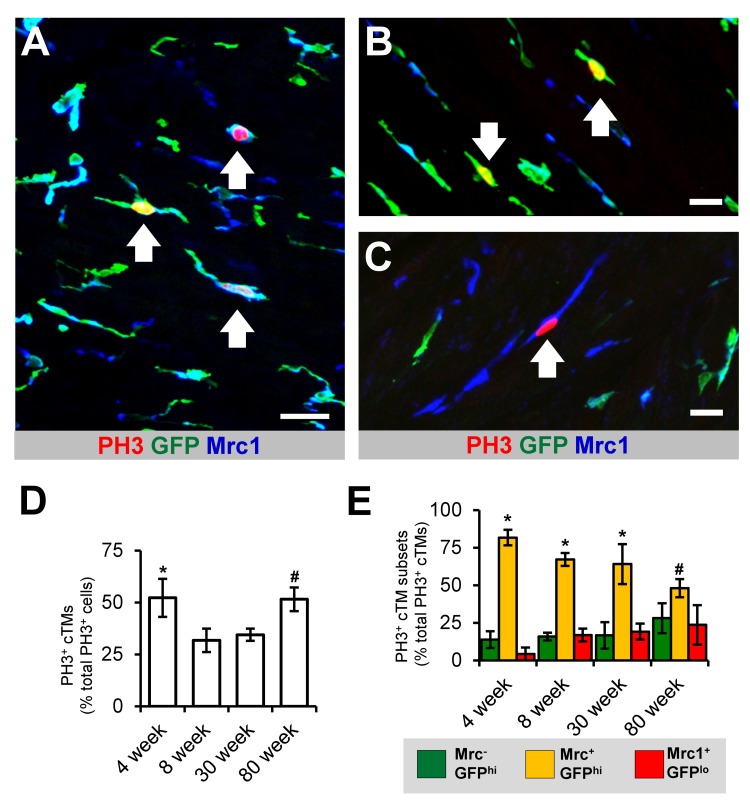
Proliferative potential of cTMs **(A)** 45 μm confocal micrograph maximum intensity view of GFP^hi^, Mrc1^+^ and PH3^+^ cells within the heart of an 8 week old *Cx_3_cr1^GFP/+^* mouse heart. **(B and C)** Magnified view of PH3^+^ Mrc1^−^GFP^hi^ and Mrc1^+^GFP^lo^ cTMs, respectively. Scale bars indicates 30 μm (A) and 20 μm (B and C). Arrows indicate PH3^+^ cTMs. **(D)** Proportion of total cardiac PH3^+^ nuclei comprised of PH3^+^ cTM nuclei. *p>0.05 4 week old vs all other ages; #*p*>0.05 80 week old vs 8 or 30 week old. **(E)** Proportion of total PH3^+^ cTM nuclei comprised of Mrc1^−^GFP^hi^, Mrc1^+^GFP^hi^ or Mrc1^+^GFP^lo^ cTM nuclei. *p≤0.05 Mrc1^+^GFP^hi^ vs Mrc1^+^GFP^lo^ or Mrc1^−^GFP^hi^; *p>0.05 Mrc1^+^GFP^hi^ vs Mrc1^+^GFP^lo^ or Mrc1^−^GFP^hi^. All histograms show means ± SEM. PH3^+^ cell numbers were determined from 3 mouse hearts/age group from multiple fields of view

To determine whether the density and distribution of GFP^+^ cTMs changes with age, we conducted confocal microscopy on cardiac sections from *Cx_3_cr1^GFP/+^* mice aged 4, 8 or 30 weeks (Fig. 3A). All three cTM subsets were present at all ages, including Mrc1^+^GFP^lo^ cTMs that in confocal micrographs appear GFP^−^ (Supplemental Fig. 3). To quantify the reduction of GFP^+^ cTMs, we measured the proportion of nuclei corresponding to GFP^+^ and non-GFP^+^ cells, which decreased from approximately 13.3% and 13.9% (in 4 and 8 week old mice respectively) to 8.0% at 30 weeks (Fig. 3B). Intriguingly, a redistribution of cTM subsets was observed throughout the myocardium of older hearts (Fig. 4A a' and a'a'), with decreased GFP^hi^ cells in the epicardium (Fig. 3C).

Recent reports have indicated that macrophages have significant replicative potential in response to inflammation and in steady state [5]. To determine the proliferative capacity of cTMs and whether this changes with age, we quantified the total number of cTMs staining for the proliferation antigen phospho histone H3 (PH3) in the mouse heart (Fig. 4). cTMs comprised 52.2% of all PH3^+^ cells in young mice (4 week old mice) (Fig. 4D), declining to 31.8% and 34.4% in 8 and 30 week old mice respectively, thereafter increasing to 51.5% in 80 week old animals (Fig. 4D), possibly due to reduction in proliferation of non-cTMs. The PH3^+^ cTMs exhibited a heterogeneous macrophage morphology, with and without cellular projections (Supplemental Video 1). Comparing the proliferative capacity of the three cTM subsets revealed that Mrc1^+^GFP^+^ cells contributed the greatest proportion of PH3^+^ cTMs in all age groups analyzed (Fig. 4E). Indeed, cTM cell division continued in senile mouse hearts (>100 weeks old; Supplemental Fig. 4). Thus, cTMs contribute to a significant proportion of dividing cells in the murine heart and retain their proliferative potential through old age (80 week old animals) and senescence.

### Gene expression differences in Mrc1^+^ cTM subsets

**Figure 5:**
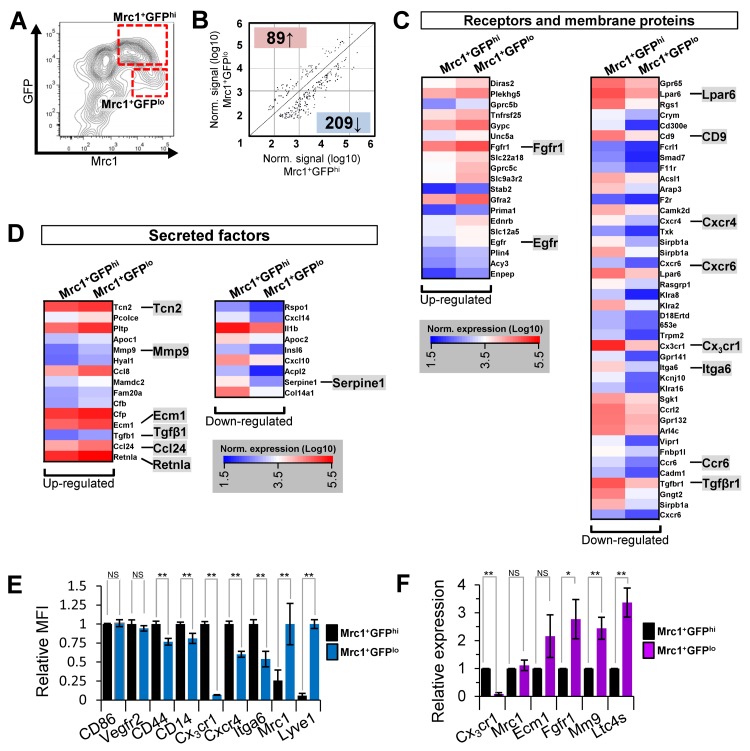
Microarray gene expression analysis of Mrc1^+^GFP^hi^ and Mrc1^+^GFP^lo^ cTM subsets and validation **(A)** Flow cytometry contour plot of cardiac myeloid (CD45^+^CD11b^+^) cells from *Cx_3_cr1^GFP/+^* mouse hearts with cTMs isolated for microarray analysis indicated (red dotted boxes). **(B)** Scatter plot of Mrc1^+^GFP^hi^ and Mrc1^+^GFP^lo^ gene probes ≥2.0-fold enriched (*p*≤0.05). Number of genes up-regulated or down-regulated in Mrc1^+^GFP^lo^ cTMs relative to Mrc1^+^GFP^hi^ cTMs are highlighted red or blue. **(C)** Receptors and membrane proteins up- or down-regulated in Mrc1^+^GFP^lo^ cTMs relative to Mrc1^+^GFP^hi^ cTMs. **(D)** Secreted factors up- or down-regulated in Mrc1^+^GFP^lo^ cTMs relative to Mrc1^+^GFP^hi^ cTMs. **(E)** Flow-cytometry analysis of differentially expressed cell surface markers (*n*=5 per marker). MFI = mean fluorescence intensity. **(F)** qRT-PCR analysis of differentially expressed genes (*n*≥4 per gene). NS = non-significant; *p<0.05; **p<0.01

Gene expression profiles of Mrc1^+^GFP^hi^ and Mrc1^+^GFP^lo^ cTMs from 15-25 week old *Cx_3_cr1^GFP/+^* mice were compared by Agilent microarray analysis (Fig. 5A). Archetypical macrophage-related genes, including colony stimulating factor 1 receptor (Csf1r), Lysozyme 1 (Lyz1), and cluster differentiation antigen 68 (CD68) were highly enriched in both cTM subsets, in addition to housekeeping genes β-actin (ActB) and glyceraldehyde-3-phosphate dehydrogenase (Gapdh) (Supplemental Fig. 5). While global gene expression patterns were similar between the two subsets (R=0.974; Supplemental Fig. 5), statistical analysis identified 298 probes with greater than 2-fold expression differences (p≤0.05; Fig. 5B; Supplemental Table 1). In addition to Cx_3_cr1, expression of a range of genes was reduced in Mrc1^+^GFP^lo^ cells, including cell surface protein genes lysophosphatidic acid receptor 6 (Lpar6), cluster of differentiation 9 (CD9), chemokine (c-x-c motif) receptor 4 (Cxcr4), chemokine (c-x-c motif) receptor 6 (Cxcr6), integrin α6 (Itga6), chemokine (c-c motif) receptor 6 (Ccr6) and transforming growth factor beta receptor 1 (Tgfβr1). Conversely, in Mrc1^+^GFP^lo^ cTMs, a number of genes were up-regulated including endothelial growth factor receptor (Egfr) and fibroblast growth factor receptor 1 (Fgfr1; Fig. 5C). Various secreted proteins were also increased in expression in Mrc1^+^GFP^lo^ cTMs, most notably, transforming growth factor β1 (Tgfβ1; expressed at relatively low amounts in both Mrc1^+^ cTM subsets), extracellular matrix protein 1 (Ecm1), procollagen C-endopeptidase enhancer (Pcolce), matrix metallopeptidase 9 (Mmp9), Transcobalamin II (Tcn2), resistin-like molecule alpha (Retnla), chemokine (C-C motif) ligand 24 (Ccl24) and chemokine (C-C motif) ligand 8 (Ccl8) (Fig. 5D). Intriguingly, leukotriene c4 synthase (Ltc4s), Thymosin β 4 (Tmsβ4x) and CCAAT/enhancer binding protein beta (Cebpβ), recently implicated in tissue regeneration [13-16], were amongst the most highly expressed genes in both macrophage subsets (Supplemental Fig. 6).

**Figure 6:**
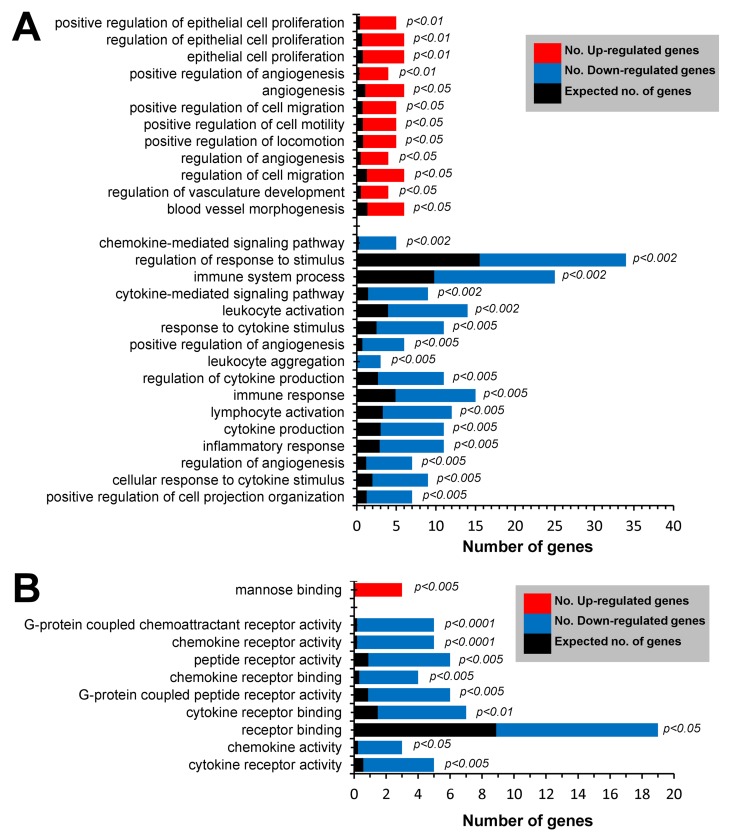
GO enrichment analysis of differentially expressed genes in Mrc1^+^ cTMs ≥2.0 fold up-regulated (p≤0.05) or ≥2.0-fold down-regulated (p≥0.05) in Mrc1^+^GFPlo cTMs relative to Mrc1^+^GFPhi cTMs (red and blue columns respectively). **(A)** Enrichment of *biological process* terms in differentially expressed gene subsets. **(B)** Enrichment of *molecular process* terms in differentially expressed gene subsets

Gene Ontology (GO) enrichment analysis of genes down-regulated (≤2.0-fold, p≤0.05) in Mrc1^+^GFP^lo^ cTMs yielded biological process GO terms such as *regulation of response to stimulus*, *leukocyte activation*, *response to cytokine stimulus*,and *leukocyte aggregation*, consistent with the loss of Cx_3_cr1 gene expression (Fig. 6A). Genes up-regulated (≥2.0-fold, p≤0.05) in Mrc1^+^GFP^lo^ cTMs were also enriched in GO terms such as*positive regulation of epithelial cell proliferation*, and *positive regulation of cell migration* (Fig. 6A). *Mannose binding* was the only molecular function GO term significantly enriched in genes up-regulated in Mrc1^+^GFP^lo^ cTMs (Fig. 6B).

Flow cytometry and quantitative real-time PCR (qRT-PCR) analyses of Mrc1^+^GFP^hi^ and Mrc1^+^GFP^lo^ cTMs validated genes that were highly expressed in both subsets (cluster of differentiation 86 (CD86), vascular endothelial growth factor receptor 2 (Vegfr2), cluster of differentiation 44 (CD44) and CD14; Fig. 5E). Genes with reduced expression in Mrc1^+^GFP^lo^ cTMs compared to Mrc1^+^GFP^hi^ cTMs included Cx_3_cr1, Cxcr4, and Itga6. Expression of CD44 and CD14 was more modestly decreased in Mrc1^+^GFP^lo^ cTMs, consistent with the loss of injury and/or foreign antigen response genes. Expression of Mrc1 and Lyve1 was increased in Mrc1^+^GFP^lo^ compared to Mrc1^+^GFP^hi^ cTMs (Fig. 5E), as well as Fgfr1, Mmp9 and Ltc4s. (Fig. 5F). Notably, qRT-PCR analysis revealed similar gene transcript levels of Mrc1 in both cTM subsets (Fig. 5), contrasting with flow cytometric data and pointing to the possible post-transcriptional regulation of Mrc1 protein levels at the cell surface.

## DISCUSSION

The aging heart is increasingly vulnerable to irreversible damage following cardiac injury. As with humans, younger mice respond to cardiac injury with improved prognosis compared to aged mice [17,18]. In this study, we provide evidence for the contribution of tissue macrophages to relatively early changes in the heart that may anticipate age-related increases in cardiac susceptibility. Although cTMs proliferate throughout life, alterations in their composition, gene expression, and function precede increased cardiac fibrosis and impairments in the inflammatory response typical of the aging heart. Our findings provide new insights into the impact of age on shifts in cTMs phenotype, suggesting that cardiac senescence is heralded by changes in resident immune cell composition.

Recent reports have indicated that local macrophage proliferation is a major mechanism of tissue macrophage turn-over in steady state and after inflammation [5, 7]. In the heart, we found cTMs comprise approximately 30-50% of all mitotic cells within the age range of 4 to 80 weeks. This observation suggests that cTM proliferation, in particular proliferation of Mrc1^+^GFP^hi^ cTMs, may be a significant source of cTM colonization in the mouse heart that persists with aging.

While Mrc1^−^GFP^hi^ and Mrc1^+^GFP^hi^ cTM subsets are present throughout life, an additional subset, Mrc1^+^GFP^lo^, which emerges relatively early in the mature heart, provides important clues to altered cardiac function. Mrc1^+^ cTMs are likely the principal myeloid environmental sentinels in the mouse heart as only Mrc1^+^ cells are macropinocytic, whereby MPs indiscriminately sample the local microenvironment for tissue damage and pathogen signatures before presentation of antigens to T-cells via MHCII molecules [9, 19]. Consistent with this finding, only Mrc1^+^ cTMs are MHCII^+^, CD64^+^ and CD14^+^, indicating that they undertake antigen presentation and detect antibody-bound targets and tissue damage signatures. Indeed, removal of early apoptotic cells is dependent on both CD14 and macropinocytosis [20].

Differences in gene expression between the two Mrc1^+^ cTM subsets are consistent with diminished responses of Mrc1^+^GFP^lo^ cTMs to tissue damage, suggesting that they are less sensitive to inflammatory stimuli with reduced contribution to the extracellular milieu [21, 22]. This is consistent with the observed decreases in CD14 and CD44 levels, both of which are important for detection of tissue damage and cell mobilization.

The loss of Cx_3_cr1 in aging cTMs predicts a functional impairment in their capacity to home and align with Cx_3_cl1 gradients that are established in mice and humans after MI [23]. As Cx_3_cr1 expression is important for macrophage activation and contribution to the inflammatory milieu [22], loss of injury response receptors such as Cx_3_cr1 in cTMs of aging hearts may affect the inflammatory milieu and extrinsic leukocyte infiltration, in addition to muting chemotactic responses.

Much enthusiasm has been generated regarding the potential for regulating epicardial inflammatory signaling and cell activation to improve cardiac repair and even regenerate the heart following injury. In this context, age-dependent change in cTM injury responses is particularly relevant since epicardial cTMs in aged hearts are exclusively Mrc1^+^GFP^lo^, and analysis of down-regulated genes in aged injured hearts underscores a corresponding change in cTM-derived epicardial signaling. As modulation of epicardial signaling or paracrine activity can affect cardiac repair and the severity of injury [14, 24] the age-dependent gene expression changes occurring in epicardial Mrc1^+^ cTMs may affect epicardial inflammatory signaling after injury, stem/progenitor cell activation and tissue repair.

Differentially expressed genes in Mrc1^+^ cTMs also point to the potential role of Mrc1^+^GFP^lo^ cTMs in the accumulation of cardiac fibrosis in the aging heart. Fibrosis is a hallmark of cardiac senescence and is associated with stiffening of the heart and impaired function [25], although the molecular and cellular bases for the accumulation of cardiac fibrosis remain unclear [26]. Age-dependent emergence of Mrc1^+^GFP^lo^ cTMs may potentiate the heart towards fibrosis by the up-regulation of genes implicated in the accumulation of cardiac fibrosis such as Fgfr1 [27], Ltc4s [28-31], Mmp9 [32], Retnla [33, 34], and Ccl24 [35]. Accordingly, these observations support the ‘hyperfunction’ model of tissue aging, which postulates that a major mechanism of tissue aging is persistent growth signalling [36, 37]. In this context, our findings suggest that cTM-derived paracrine factors may play a prominent role in promoting cardiac aging.

Conclusive demonstration that age-dependent cTM changes lead to functional deficits or impaired cardiac injury responses awaits approaches that permit organ-specific manipulation of cTMs (currently unavailable), or identification of factors that regulate gene signatures of cTM subsets. The current analysis has been to aging-dependent changes in cTMs in the C57BL6 inbred mouse line, and strain differences in cTM gene expression remain to be explored. Finally, this work prompts investigation of potential gene expression changes in tissue macrophages of non-cardiac tissues, as Cx_3_cr1 is expressed in subsets of MPs and natural killer cells in a range of organs [38]. The extent to which similar aging-dependent gene alterations in Cx_3_cr1 and other genes takes place in tissue MPs in other organs warrants investigation.

In summary, age-dependent changes in cTM gene expression point to early mechanisms underlying cardiac senescence, related mortality and morbidity following cardiac injury. Whether cTM subsets can be manipulated to improve cardiac repair after injury by activating epicardial and other endogenous cardiac progenitors remains to be explored. Given the challenges to ensure therapeutic cell retention and functional integration in the context of cardiac damage, this abundant and proliferative resident cardiac cell subset represents a novel target for beneficial manipulation of the cardiac milieu in stem cell therapeutic contexts.

## METHODS

### Mice

All mice were maintained in the C57BL/6 background in specific pathogen-free (SPF) environment and fed standard mouse diet *ad libitum*. *Cx_3_cr1^GFP/+^*transgenic mice were a gift from C. Gross (European Molecular Biology Laboratory, Montero-tondo, Italy). All procedures conducted were approved by the Monash University Animal Research Platform 2 (MARP2) Animal Ethics Committee.

### Preparation of single cells and flow cytometry

Cardiac single cells preparations were conducted as described in detail previously [39]. Briefly, mice were killed by CO2 asphyxiation (~5 min) followed by cardiac perfusion with ice-cold HBSS for 2 min. Hearts were isolated and atria and coronary valves removed before being minced with surgical scissors. Cells were dissociated from minced tissue by using gentleMACS Dissociator (Milenyi Biotec, North Ryde, Australia) according to manufacturer's protocol for mouse heart processing. Cell/tissue suspensions were filtered through 70 μm filters, centrifuged and resuspended 2% fetal bovine serum (FBS)/PBS until further processing.

### Immunostaining and confocal microscopic analysis

For preparation of cardiac tissue for immunostaining, mice were killed by CO_2_ asphyxiation (~5 min) followed by cardiac perfusion with first, ice-cold HBSS for ~2 min and then freshly prepared ice-cold 4% formaldehyde/phosphate buffered saline (PBS; ~5 min) through the left ventricle and tissue was harvested and incubated in fresh 4% formaldehyde/PBS overnight. For thick section staining, tissue was sectioned using a vibrating blade microtome (Leica Microsystems, North Ryde, Australia) at 150 μm/section. Prepared sections were permeabilized with 0.2% Triton X-100 (Sigma-Aldrich, Castle Hill, Australia)/PBS solution, before blocking in 1% goat-serum/0.2% Triton X-100/PBS. Standard immunostaining protocols were used for following steps. The primary antibodies used for staining of sections are summarized in Supplemental Table 2. Alexa Fluor conjugated secondary antibodies (Life Technologies, Mulgrave, Victoria) were used for fluorescence staining. Fluorescent microscopy images were obtained using a Leica SP5 confocal laser scanning microscope (Leica Microsystems, North Ryde, Australia) and a Nikon C1 confocal laser scanning microscope (Nikon Instruments, Melville, New York, USA). Two and three-dimensional images were prepared using Imaris software (Bitplane, Zurich, Switzerland) or FIJI software[40], with adjustments made to brightness and contrast.

Quantification of cells in microscopy images were conducted using Imaris software. To quantify the number of GFP^+^ nuclei within the myocardium, nuclei were registered based on DAPI fluorescence intensity and proportion of nuclei that are GFP^+^ determined. To enumerate the proportion of cTM subsets at the epicardium, cTM subsets at the epicardium (external surface of the cardiac section) were manually counted based on GFP and/or Mrc1 fluorescence. Similarly, PH3^+^ cells were manually counted based on PH3, GFP and Mrc1 fluorescence from heart sections from *Cx_3_cr1^GFP/+^* mice at different ages.

### Flow cytometry and cell sorting

The antibodies used for staining of single cell suspensions after FC receptor blocking with CD16/CD32 are summarized in Supplemental Table 2. Cells were analyzed by a LSR II Flow Cytometer (BD Biosciences, North Ryde, Australia) using FlowJo Software (Tree Star, Ashland, Oregon, USA). Fluorescence activated cell sorting (FACS) was conducted using BD Influx cell sorters. All sorted cells were isolated into lysis buffer provided by the RNeasy Micro kit (Qiagen, Chadstone, Australia).

### In vivo cTM macropinocytosis and phagocytosis

Macropinocytosis was detected using 2MDa TRITC-labelled dextran (TRITC-dextran) as previously described[1]. Briefly, 50 μg TRITC-dextran was injected intravenously to 15-25 week old mice in 200 μl pre-warmed HBSS. 20 hours after injection cardiac cells were analysed by flow cytometry for TRITC fluorescence as described above. DiI liposome phagocytosis was determined as for the macropinocytosis experiment, except 200 μl of *Fluoroliposome™* solution (DiI-loaded liposomes; Encapsula Nanosciences, Brentwood, Tennessee, USA) was used and cells analyzed 24 hours after injection.

### cTM isolation for gene expression analysis

cTMs were isolated by collagenase digestion as previously described [39]. For RNA analysis, cTMs were enriched from cardiac cell preparations using paramagnetic CD45-microbeads and sorted directly into cell lysis buffer provided by the RNA extraction kit by fluorescence activated cell sorting (FACS) before RNA extraction. RNA extracts were used for quantitative real time PCR (qRT-PCR) following reverse transcription to generate cDNA, or microarray analysis (as described below).

### Quantitative Real Time PCR (qRT-PCR)

RNA was isolated as described above. RNA quality was assessed by spectrophotometry using a NanoDrop ND-1000 (Thermo Fisher, Scoresby, Australia). Reverse transcription was performed using the SuperScript VILO cDNA Synthesis Kit (Life Technologies, Mulgrave, Australia). Quantitative PCR assays were performed using LightCycler 480 SYBR green (Roche Diagnostics, Castle Hill, Australia). Gene expression levels were calculated using the 2^−ΔΔCt^ method using the geometric mean of at least 2 housekeeping genes as normalizers. Primer sequences used in qRT-PCR gene expression analysis are listed in Supplemental Table 3.

### Microarray analysis

Mrc1^+^ cTM subsets (CD45^+^CD11b^+^Mrc1^+^GFP^hi^ and CD45^+^CD11b^+^Mrc1^+^ GFP^lo^) were isolated by FACS (as described above). Total RNA samples were isolated from three independent biological samples using the RNeasy Micro kit (Qiagen, Chadstone, Australia). Amplified RNA (aRNA) was produced from isolated RNA from cTMs using the Arcturus RiboAMP PLUS RNA amplification kit (Life Technologies, Mulgrave, Australia). aRNA was analyzed with a Bioanalyzer (Agilent Technologies, Mulgrave, Australia) system for RNA quality. 2 μg of aRNA was Cy3-labelled using Kreatech ULS Fluorescent Labelling Kit (Kreatech Diagnostics, Amsterdam, The Netherlands) and 600 ng of labelled aRNA was analyzed on a Mouse Gene Expression − 8 × 60K G4852A microarray chip (Agilent Technologies, Mulgrave, Australia). All processing of aRNA samples was conducted by the Monash Health Translation Precinct Medical Genomics Facility. For gene expression analysis, data was extracted and normalized (quantile) using Subio Platform software (Subio Inc., Amami-shi, Japan). Probes were filtered on flags and to include those where at least 1 of 3 samples had a normalized intensity value in the top 80% of probes based on normalized signal intensity. Datasets were derived from three biologically independent replicate samples. In comparative analyses, probes were filtered to include those that 2-fold or greater differences with *p*≤0.05 (Student's T-test with Bonferroni adjustment). All microarray data files are available at ArrayExpress database (www.ebi.ac.uk/arrayexpress) under the accession number E-MTAB-2168.

### Gene Ontology (GO) enrichment analysis

GO enrichment analysis was conducted on differentially expressed genes using the online tool WebGestalt (http://bioinfo.vanderbilt.edu/webgestalt/)[41].

Hypergeometric statistical method with Benjamini and Hochberg multiple test adjustment was applied.

### Statistical analyses

Student's T-Test (two-tailed, assuming normal distribution) was applied using Microsoft Excel software for all statistical analyses, except those for microarray gene expression profiling and GO enrichment analysis (see respective detailed methods sections above). For all statistical tests, differences were considered statistically significant where *p*≤0.05.

## SUPPLEMENTAL FIGURES, TABLES and VIDEOS


