# Supplementary material for: Age-related changes in tissue macrophages precede cardiac functional impairment
Source: Aging (Albany NY). 2014 May 23;6(5):399–413. doi: 10.18632/aging.100669 (PMC4069267; doi:10.18632/aging.100669)
Supplement: Supplementary file 1 [file aging-06-399-s001.pdf]

SUPPLEMENTAL FIGURES

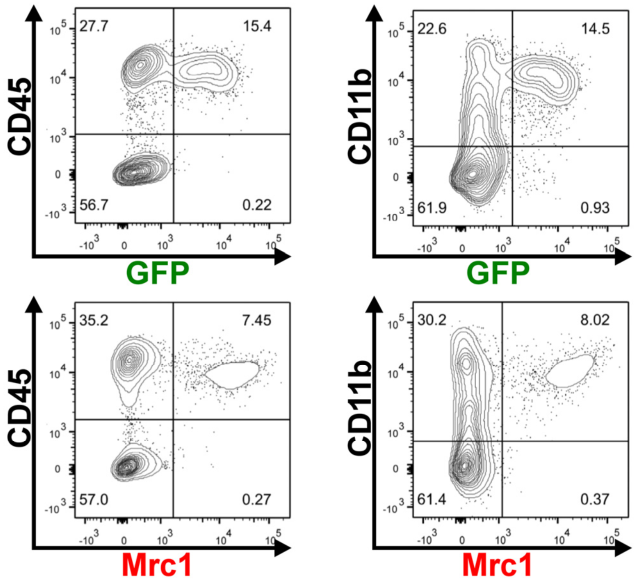

Supplemental Figure 1. CD45 and CD11b expression in GFP$^+$ and Mrc1$^+$ cells from *Cx$_3$cr1$^{GFP/+}$* mouse hearts. (Top panels) GFP expression relative to CD45 (top right) and CD11b (top left). **(Bottom panels)** Mrc1 expression relative to CD45 (bottom right) and CD11b (bottom left). All panels were gated on viable events identified by exclusion of viability dye. Quadrant numbers indicate percentage of total events. Data representative multiple similar experiments.

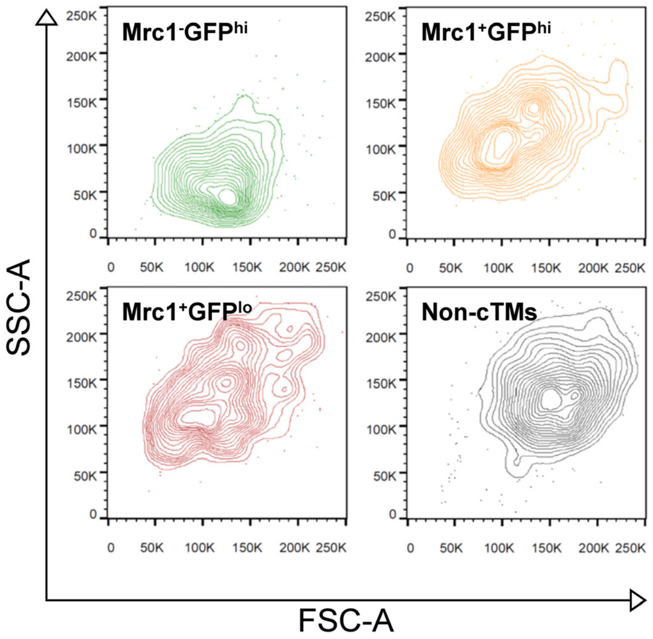

Supplemental Figure 2. Representative scatter profile of cardiac myeloid cells. Forward scatter-area (FSC-A) and side scatter-area (SSC-A) of cardiac myeloid cells (CD45$^+$CD11b$^+$). Scatter profiles of the three cTM subsets (Mrc1$^-$GFP^{hi}, Mrc1$^+$GFP^{hi} and Mrc1$^+$GFP^{lo}) and non-cTMs (Mrc1$^-$GFP$^-$) are shown individually as indicated.

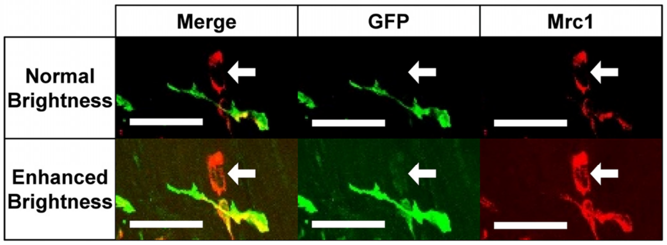

Supplemental Figure 3. Mrc1$^+$GFP^{lo} cells express GFP although appearing GFP$^-$. Arrows indicated an Mrc1$^+$GFP^{lo} cTM. Without image adjustment ('normal brightness', top panels), Mrc1$^+$GFP^{lo} cTMs appear GFP$^-$. Upon brightness enhancement ('enhanced brightness', bottom panels), GFP signal is visible.

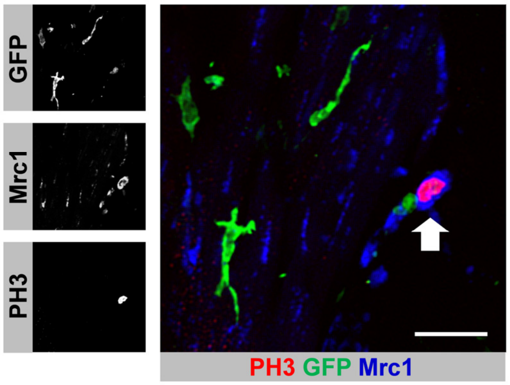

Supplemental Figure 4. Dividing cTMs in a 104 week-old *Cx$_3$cr1$^{GFP/+}$* mouse heart. PH3$^+$ nuclei (red), GFP (green) and Mrc1 (blue) with PH3$^+$Mrc1$^+$GFP^{lo} cTM indicated (arrow). Scale bar indicates 30 µm. Miniature panels (left) show fluorescence signals for individual markers as indicated.
